# Supplementary material for: A fault-intrusion-tolerant system and deadline-aware algorithm for scheduling scientific workflow in the cloud
Source: PeerJ Comput Sci. 2021 Nov 2;7:e747. doi: 10.7717/peerj-cs.747 (PMC8576554; doi:10.7717/peerj-cs.747)
Supplement: Supplemental Information 6 [file peerj-cs-07-747-s006.zip › Myproject1.1/docs/org/workflowsim/utils/Parameters.html]

Parameters


JavaScript is disabled on your browser.


- Overview
- Package
- Class
- Use
- Tree
- Deprecated
- Index
- Help

- Prev Class
- Next Class

- Frames
- No Frames

- All Classes

- Summary:
- Nested |
- Field |
- Constr |
- Method

- Detail:
- Field |
- Constr |
- Method


org.workflowsim.utils

## Class Parameters

- java.lang.Object
- - org.workflowsim.utils.Parameters

- ---

    

  ```
  public class Parameters
  extends java.lang.Object
  ```

  This class includes most parameters a user can specify in a configuration
  file

  Since:
  :   WorkflowSim Toolkit 1.0

- - ### Nested Class Summary

    Nested Classes

    | Modifier and Type | Class and Description |
    | --- | --- |
    | `static class` | `Parameters.FTCFailure` |
    | `static class` | `Parameters.FTCMethod` Fault Tolerant Clustering method |
    | `static class` | `Parameters.FTCMonitor` |
    | `static class` | `Parameters.PLNMethod` Planning Algorithm (Global Scheduling Algorithm) |
    | `static class` | `Parameters.SCHMethod` |
  - ### Field Summary

    Fields

    | Modifier and Type | Field and Description |
    | --- | --- |
    | `static int` | `BASE` |
  - ### Constructor Summary

    Constructors

    Constructor and Description

    `Parameters()`
  - ### Method Summary

    Methods

    | Modifier and Type | Method and Description |
    | --- | --- |
    | `static java.util.Map` | `getAlpha()` Gets the task failure rate |
    | `static java.util.Map` | `getBeta()` Gets the job failure rate (not supported yet) |
    | `static ClusteringParameters` | `getClusteringParameters()` Gets the clustering parameters |
    | `static java.lang.String` | `getDatasizePath()` Gets the data size path |
    | `static java.lang.String` | `getDaxPath()` Gets the DAX path |
    | `static long` | `getDeadline()` |
    | `static Parameters.FTCFailure` | `getFailureGeneratorMode()` Gets the failure generation mode |
    | `static Parameters.FTCMethod` | `getFTCMethod()` Gets the fault tolerant clustering method |
    | `static Parameters.FTCMonitor` | `getMonitorMode()` Gets the fault tolerant clustering monitor mode |
    | `static OverheadParameters` | `getOverheadParams()` Gets the overhead parameters |
    | `static Parameters.PLNMethod` | `getPlannerMode()` Gets the planning method |
    | `static java.lang.String` | `getReduceMethod()` Gets the reducer mode |
    | `static java.lang.String` | `getRuntimePath()` Gets the runtime file path |
    | `static Parameters.SCHMethod` | `getSchedulerMode()` Gets the scheduling method |
    | `static java.lang.String` | `getVersion()` Gets the version |
    | `static int` | `getVmNum()` Gets the vm number |
    | `static void` | `init(Parameters.FTCMethod fMethod, Parameters.FTCMonitor monitor, Parameters.FTCFailure failure, java.util.Map failureList, int vm, java.lang.String dax, java.lang.String runtime, java.lang.String datasize, OverheadParameters op, ClusteringParameters cp, Parameters.SCHMethod scheduler, Parameters.PLNMethod planner, java.lang.String rMethod, long dl)` A static function so that you can specify them in any place |
    | `static void` | `printVersion()` |
    | `static void` | `setVmNum(int num)` Sets the vm number |

    - ### Methods inherited from class java.lang.Object

      `clone, equals, finalize, getClass, hashCode, notify, notifyAll, toString, wait, wait, wait`

- - ### Field Detail


    - #### BASE

      ```
      public static final int BASE
      ```

      See Also:
      :   Constant Field Values
  - ### Constructor Detail


    - #### Parameters

      ```
      public Parameters()
      ```
  - ### Method Detail


    - #### init

      ```
      public static void init(Parameters.FTCMethod fMethod,
              Parameters.FTCMonitor monitor,
              Parameters.FTCFailure failure,
              java.util.Map failureList,
              int vm,
              java.lang.String dax,
              java.lang.String runtime,
              java.lang.String datasize,
              OverheadParameters op,
              ClusteringParameters cp,
              Parameters.SCHMethod scheduler,
              Parameters.PLNMethod planner,
              java.lang.String rMethod,
              long dl)
      ```

      A static function so that you can specify them in any place

      Parameters:
      :   `fMethod,` - the fault tolerant clustering method
      :   `monitor,` - the fault tolerant clustering monitor
      :   `failure,` - the failure generation mode
      :   `failureList,` - the task failure list
      :   `vm,` - the number of vms
      :   `dax,` - the DAX path
      :   `runtime,` - optional, the runtime file path
      :   `datasize,` - optional, the datasize file path
      :   `op,` - overhead parameters
      :   `cp,` - clustering parameters
      :   `scheduler,` - scheduling mode
      :   `planner,` - planning mode
      :   `rMethod` - , reducer mode
      :   `deadline,` - deadline of a workflow


    - #### getOverheadParams

      ```
      public static OverheadParameters getOverheadParams()
      ```

      Gets the overhead parameters

      Returns:
      :   the overhead parameters


    - #### getAlpha

      ```
      public static java.util.Map getAlpha()
      ```

      Gets the task failure rate

      Returns:
      :   the task failure rate


    - #### getBeta

      ```
      public static java.util.Map getBeta()
      ```

      Gets the job failure rate (not supported yet)

      Returns:
      :   the job failure rate


    - #### getReduceMethod

      ```
      public static java.lang.String getReduceMethod()
      ```

      Gets the reducer mode

      Returns:
      :   the reducer


    - #### getFailureGeneratorMode

      ```
      public static Parameters.FTCFailure getFailureGeneratorMode()
      ```

      Gets the failure generation mode

      Returns:
      :   the failure generation mode


    - #### getMonitorMode

      ```
      public static Parameters.FTCMonitor getMonitorMode()
      ```

      Gets the fault tolerant clustering monitor mode

      Returns:
      :   the fault tolerant clustering monitor mode


    - #### getDaxPath

      ```
      public static java.lang.String getDaxPath()
      ```

      Gets the DAX path

      Returns:
      :   the DAX path


    - #### getRuntimePath

      ```
      public static java.lang.String getRuntimePath()
      ```

      Gets the runtime file path

      Returns:
      :   the runtime file path


    - #### getDatasizePath

      ```
      public static java.lang.String getDatasizePath()
      ```

      Gets the data size path

      Returns:
      :   the datasize file path


    - #### getFTCMethod

      ```
      public static Parameters.FTCMethod getFTCMethod()
      ```

      Gets the fault tolerant clustering method

      Returns:
      :   the fault tolerant clustering method


    - #### getVmNum

      ```
      public static int getVmNum()
      ```

      Gets the vm number

      Returns:
      :   the vm number


    - #### setVmNum

      ```
      public static void setVmNum(int num)
      ```

      Sets the vm number

      Parameters:
      :   `vmNum` -


    - #### getClusteringParameters

      ```
      public static ClusteringParameters getClusteringParameters()
      ```

      Gets the clustering parameters

      Returns:
      :   the clustering parameters


    - #### getSchedulerMode

      ```
      public static Parameters.SCHMethod getSchedulerMode()
      ```

      Gets the scheduling method

      Returns:
      :   the scheduling method


    - #### getPlannerMode

      ```
      public static Parameters.PLNMethod getPlannerMode()
      ```

      Gets the planning method

      Returns:
      :   the planning method


    - #### getVersion

      ```
      public static java.lang.String getVersion()
      ```

      Gets the version

      Returns:
      :   version


    - #### printVersion

      ```
      public static void printVersion()
      ```


    - #### getDeadline

      ```
      public static long getDeadline()
      ```


- Overview
- Package
- Class
- Use
- Tree
- Deprecated
- Index
- Help

- Prev Class
- Next Class

- Frames
- No Frames

- All Classes

- Summary:
- Nested |
- Field |
- Constr |
- Method

- Detail:
- Field |
- Constr |
- Method
